# Onabotulinumtoxin A for Treating Overactive/Poor Compliant Bladders in Children and Adolescents with Neurogenic Bladder Secondary to Myelomeningocele

**DOI:** 10.3390/toxins5010016

**Published:** 2012-12-28

**Authors:** Antonio Marte

**Affiliations:** Pediatric Surgery 2nd University of Naples, Via Pansini 5, Naples 80131, Italy; E-Mail: antonio.marte@unina2.it; Tel./Fax: +39-815666683

**Keywords:** myelomeningocele, neurogenic bladder, vesicoureteral reflux, urodynamics, onabotulinumtoxin A

## Abstract

This retrospective study was performed to verify the efficacy and safety of Onabotulinumtoxin A (BTX-A) in treating children with neurogenic bladder (NB) secondary to myelomeningocele (MMC) with detrusor overactivity/low compliance. From January 2002 to June 2011, 47 patients out of 68 with neuropathic bladder were selected (22 females, 25 males, age range 5–17 years; mean age 10.7 years at first injection). They presented overactive/poor compliant neurogenic bladders on clean intermittent catheterization, and were resistant or non compliant to pharmacological therapy. Ten patients presented second to fourth grade concomitant monolateral/bilateral vesicoureteral reflux (VUR). All patients were incontinent despite catheterization. In the majority of patients Botulinum-A toxin was administered under general/local anesthesia by the injection of 200 IU of toxin, without exceeding the dosage of 12IU/kg body weight, diluted in 20 cc of saline solution in 20 sites, except in the periureteral areas. Follow-up included clinical and ultrasound examination, urodynamics performed at 6, 12 and 24 weeks, and annually thereafter. Seven patients remained stable, 21 patients required a second injection after 6–9 months and 19 a third injection. VUR was corrected, when necessary, in the same session after the BT-A injection, by 1–3 cc of subureteral Deflux^®^. Urodynamic parameters considered were leak point pressure (LPP), leak point volume (LPV) and specific volume at 20 cm H_2_O pressure. The results were analyzed using the Wilcoxon test. All patients experienced a significant 66.45% average increase of LPV (Wilcoxon paired rank test = 7169 × 10 ^−10^) and a significant 118.57% average increase of SC 20 (Wilcoxon paired rank test = 2.466 × 10 ^−12^). The difference between preoperative and postoperative LPP resulted not significant (Wilcoxon paired rank test = 0.8858) No patient presented severe systemic complications; 38/47 patients presented slight hematuria for 2–3 days. Two patients had postoperative urinary tract infection. All patients were hospitalized for 24 h with catheterization. Thirty-eight out of 47 patients achieved dryness between CIC; nine patients improved their incontinence but still need pads. Ten patients have resumed anticholinergic agents. Our results suggest that the use of BTX-A is safe and effective in patients with MMC with a positive effect on their dryness and quality of life.

## 1. Introduction

Current treatment of patients with neurogenic bladder (NB) secondary to myelomeningocele (MMC) is mainly based on Clean Intermittent Catheterization (CIC) and associated anticholinergic agents, or on surgical procedures on the bladder or bladder neck. In cases where the NB is characterized by overactivity and/or low compliance, the first safeguard is the use of early anticholinergic drugs, such as oxybutynin, tolterodine, or most recently trospium chloride, with the ultimate goal of making the patient dry safeguarding renal function. If anticholinergics and CIC do not provide the desired result, then it is necessary to use more invasive techniques of augmentation in order to transform the bladder into a reservoir with high capacity/low pressure [1]. Based on recent research, there is increasing confidence in the use of onabotulinumtoxin A (Botox—BTX-A) in the treatment of NB secondary to MMC as a valid alternative to invasive procedures [2,3,4,5]. We present our experience of a selected a group of patients in whom the clinical and urodynamics assumed the need for augmenting their bladder due to the poor response to drugs, and in whom incontinence and the use of pads between catheterizations strongly influenced their social lives. 

## 2. Experimental Section

This retrospective study reflects 68 patients referred to our institution from January 2002 to June 2011. Forty-seven patients out of 68 (69%) with neurogenic bladders were selected for BTX-A treatment. There were 22 females and 25 males, age range 5–17 years at first injection, with a mean age of 10.7 years. Patients possessed overactive/poorly compliant bladders on clean intermittent catheterization (CIC), and were resistant/non compliant to pharmacological therapy. Ten patients presented second to fourth grade concomitant mono/bilateral vesicoureteral reflux (VUR). Despite the treatment, the patients complained of urinary incontinence between catheterizations. All patients were regularly monitored with periodic urodynamic examinations, voiding cystourethrography and antibiotic prophylaxis when necessary. After informed consent and the approval of the ethics committee, the patients were offered the possibility of BTX-A injection before considering more invasive procedures.

As an imperative condition, there was the requirement that all the patients had negative urine cultures prior to the procedure. With patients in the lithotomy position, the injection was carried out when the bladder reached a sufficient filling to ensure a good overview, but avoiding overdistension. The administration of toxin was carried out with general or local anesthesia in more collaborative patients; in the latter, 15 min prior to the procedure, 15–25 cc of lidocaine to 0.5% was instilled into the bladder through the catheter. The solution used for injection was obtained by preparing two vials of 100 IU of BTX-A diluted in 20 cc of 0.9% saline solution for a total of 200 IU, avoiding shaking the vial too roughly and without exceeding the dosage of 12 IU /kg body weight. The injection was performed by a needle-metallic cannula of 3.7 Ch with 23 G needle of the type used for the correction of reflux or a flexible Cook needle. The needle was inserted between 3–4 mm in the bladder wall and in each site a quantity of BTX-A of 1 cc = 10 IU Botox was injected for a total of 20 injections. After a survey of endoscopic bladder and ureteral ostia was performed, BTX-A was injected according to a “blind multipoint” procedure. Injections were performed without sparing the trigone, but avoiding periureteral areas in order not to interfere with the uretero-vesical dynamics, in 20 different sites taking care not to insert the needle too deeply, especially at the level of the bladder dome to avoid inadvertently injecting the intra-abdominal viscera. The appearance of a submucosal ledge was considered a correct injection. When there were trabeculations they were preferred for the injections as they derived from a focal detrusor hypertrophy. 

**Figure 1:**
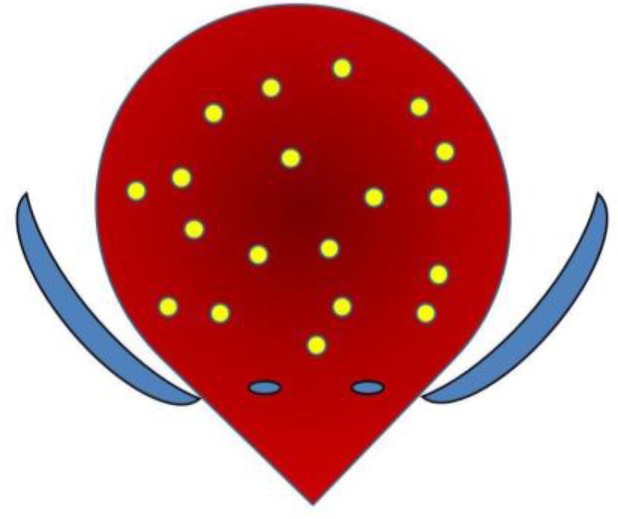
Multipoint injection scheme (1 cc = 10 IU BTX-A injected in 20 sites, sparing periureteral areas).

**Figure 2:**
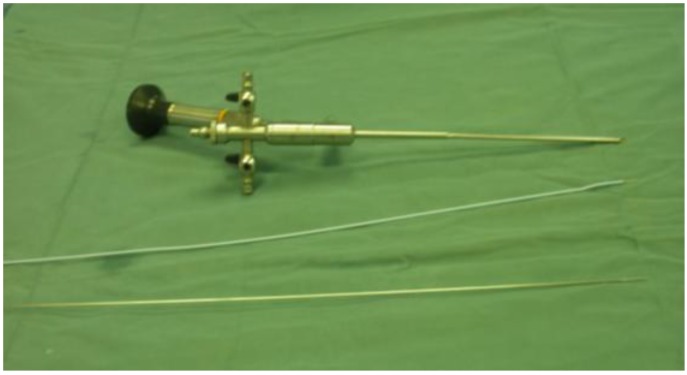
Photograph depicting equipment used to inject BT-A in this study. Cystoscope 11 Ch with a straight operative channel and the two kinds of needle were utilized (Cook needle or metallic needle utilized for endoscopic vesicoureteral reflux (VUR) treatment).

**Figure 3:**
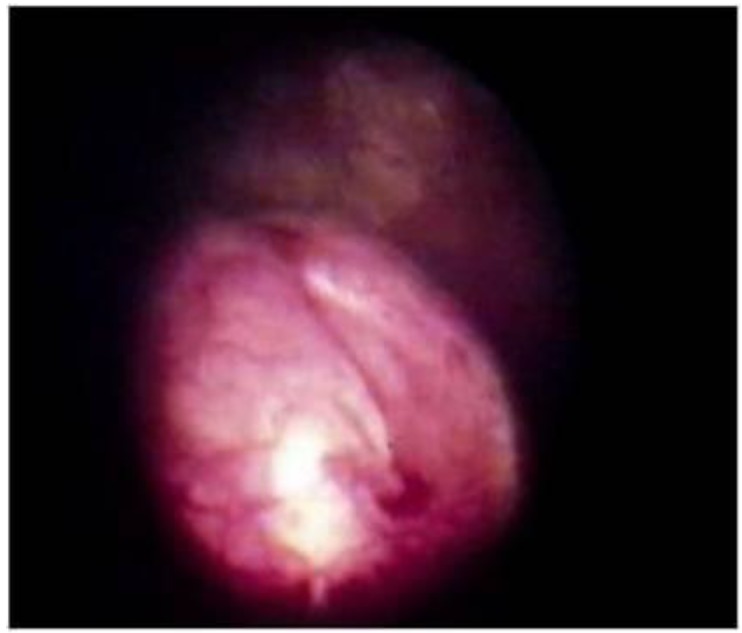
Correction of VUR in trabeculated neurogenic bladder. Notice the volcano appearance of the orifice.

A catheter was kept in place for 24 h and antibiotic was administered for five days. In patients with VUR, the correction by endoscopic subureteral injection of Deflux^®^ was performed at the end of injections utilizing 1–3 cc of bulging agent realizing the so-called TEM: Total Endoscopic Management [6]. In these latter cases, the antibiotic-prophylaxis was maintained for one month after the treatment (Figure 1, Figure 2, Figure 3).

The follow-up with ultrasound, urodynamics, and clinical evaluation mainly for the dryness was made at 6, 12 and 24 weeks. A VCUG was performed 12 weeks after the procedure in patients with VUR and reflux was considered cured if a complete resolution was obtained or a downgrading to first grade. In case of persistence, the endoscopic injection was repeated. The urodynamic parameters used were Leak Point Pressure and Leak Point Volume (LPP and LPV) and the specific bladder capacity at 20 cm H_2_O (SC 20). This index, similar to compliance, provides a useful functional datum because it indicates the ability of the bladder to fill at low pressure, within the limits of 20 cm H_2_O [7,8]. The values obtained were subjected to statistical Wilcoxon test.

## 3. Results

**Figure 4:**
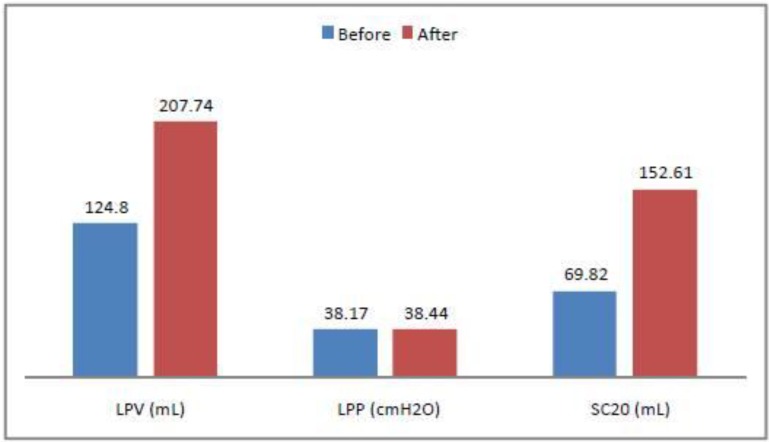
Demographic data graph before and after the first injection of BTX-A. LPV (Mean Leak Point Volume before the injection: 124.8 mL, SD 47.85423. Mean LPV after the injection 207.74 SD 57.16166), LPP (Mean Leak Point Pressure, before the injection: 38.17 SD 10.11954, after the injection: 38.44 SD 9.910049), Specific Capacity at 20 cm H2O (Mean SC before the injection: 69.82 SD 33.78147, after the injection: 152.61 SD 57.51316).

The results of this study are shown in Figure 4. LPV (Mean Leak Point Volume before the injection: 124.8 mL, SD 47.85423. Mean LPV after the injection 207.74 SD 57.16166), LPP (Mean Leak Point Pressure, before the injection: 38.17 SD 10.11954, after the injection: 38.44 SD 9.910049), Specific Capacity at 20 cm H_2_O (Mean SC before the injection: 69.82 SD 33.78147, after the injection: 152.61 SD 57.51316).

All patients experienced a significant 66.45% average increase of LPV (Wilcoxon paired rank test = 7.169 × 10^−10^) and a significant 118.57% average increase of SC 20 (Wilcoxon paired rank test = 2.466× 10^−12^). Preoperative and postoperative LPP resulted not significant (Wilcoxon paired rank test = 0.8858). 

At VCUG follow-up control, there were two recurrences of monolateral third grade reflux that required a second endoscopic treatment. All patients are still on CIC regimen; at the clinical follow-up examinations, they reported dryness between CIC with only sporadic episodes of urine leakage, and were satisfied with the results achieved. Pharmacological treatment was thus withdrawn. With the exception of seven patients (14%), which are still in clinical balance, 22 patients (46.8%) received a second and 18 (38.2%) a third injection of BTX-A after 6–9 months for the recurrence of symptoms. 

No patient presented severe systemic complications; 38/47 patients presented slight hematuria for 2–3 days. Two patients had one postoperative urinary tract infection; two patient gastric pain, treated with ranitidine; two patient facial flushing; five patients mild hypostenia of the lower limbs, resolved in 4–6 h. All patients were hospitalized for 24 h with catheterization and dismissed on day one. VUR was cured by one or two injection of Deflux. 

**Figure 5:**
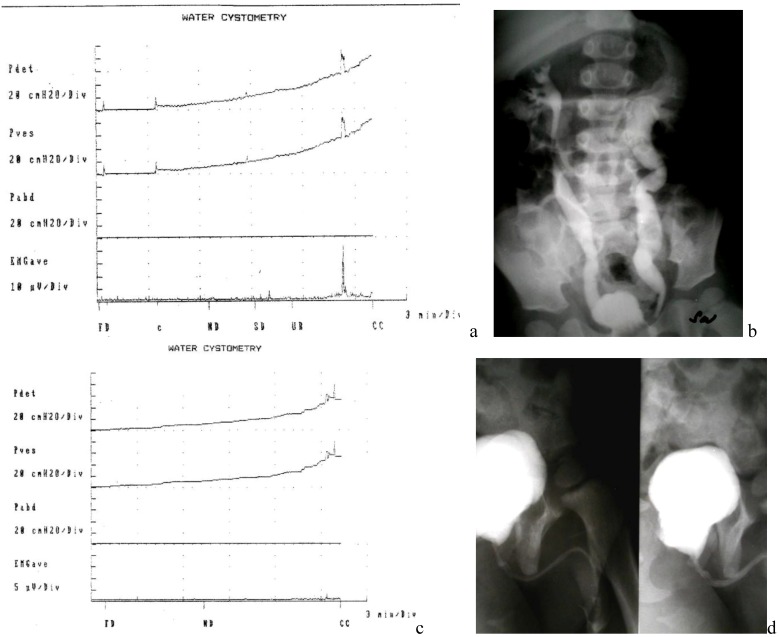
Evolution of a patient before and after BTX-A injection and endoscopic correction of VUR: (**a**) poor compliant bladder (**b**) VCUG: bilateral VUR; (**c**) improvement of bladder capacity and low pressure filling after Bt-A injection (**d**) VCUG: resolution of VUR.

After a mean follow-up of 5.7 years, 38 out of 47 patients achieved dryness between CIC even if 10 patients have resumed regularly anticholinergics, nine patients improved their incontinence but still need pads and are scheduled for a further injection of BTX-A. None of these patients nor their parents wanted to consider more invasive procedures (Figure 5).

## 4. Discussion

In our experience, a BTX-A injection is simple, effective and only mildly invasive as it does not necessarily require narcosis; it also enables the simultaneous correction of VUR to overcome the increased risk of high intravesical pressure and recurrent UTI. This treatment can decrease the incidence of renal damage in children for whom conservative management fails to help and requires only a short hospital stay; anticholinergic drugs can generally be withdrawn. The patient in this series experienced both improved LPV and specific capacity at 20 cm H_2_O. In relation to compliance, this index is especially significant as it shows satisfactory bladder filling at low pressures. However, this study shows also that the BTX-A injection only in a few cases obtains stable results (14% in our experience) with one treatment and the majority of patients will require more treatments. This should be always taken into consideration in requiring informed consent. On the other hand, the minimally invasiveness and the same repeatability are factors highly positive.

The clinical use of onabotulinumtoxin A is now a common and consolidated technique in several medical fields. Urology—specifically pediatric urology—is an example of its most recent application in neuropathic and non neuropathic patients [9]. BTX-A blocks acetylcholine release by binding, at the presynaptic level, to SNAP-25, a cytoplasmic protein on the cell membrane, which plays a major role in acetylcholine release [10,11]. The duration of the relaxing effect on the detrusor muscle is not yet known, although the repeated applications—aside from being well tolerated—seem to prolong both duration and efficacy of treatment. Another problem is the most suitable dosage in relation to the characteristics of the detrusor: in our patients, we used 200 IU BTX-A independently of age and cystometric curve. Dosage was empirically defined within a safety margin, based on other reports in the literature; therefore even if our results are satisfactory, further experience in this field will most likely help define the most appropriate toxin dosage needed [12,13,14].

Regarding the contemporary endoscopic correction of VUR in our early experience we noticed that the BTX-A alone was not effective in resolving VUR, reducing at same time the episodes of UTI and pyelonephritis. This experience is supported by similar results reported by other Authors [6,15]. We also considered that the ultimate goal of treatment of the neurogenic bladder is preserving kidneys function and a proactive treatment of risks for upper tract deterioration is mandatory. Although the small number of patients studied does not allow us to draw definite conclusions, the results show that the use of BTX-A can be suitably included in the algorithm for the treatment of neurogenic bladder and detrusoroveractivity, after anticholinergic drugs, or even the first treatment in the event of failure of drug treatment. The use of more invasive surgical operations, such as autoaugmentation or enterocystoplasty could be taken into account only in case that BTX-A treatment fails. The range of therapeutic options for this condition can safely include BTX-A treatment, along with anticholinergic drugs and traditional surgery. However, the duration of its efficacy and long-term reliability in urology should be further investigated and standardized, although the experience has shown that the technique can be safely re-administered after 6 months with good results. Moreover, no relevant side effects were detected after the administration of onabotulinumtoxin A. This also means that patients require constant clinical and instrumental controls. Another recently highlighted factor concerns the depth of the injection intradetrusor. As demonstrated by Mehnert [16] with a study by the use of MRI with toxin labeled with gadolinium, a too deep injection is deposited outside of the bladder wall. Therefore, it can be assumed that an ineffective treatment can be linked more to improper depth of the injection rather than to the dosage of toxin.

Moreover, the duration of treatment, the efficacy, and long-term reliability of the bladder remains uncertain. It is not to be underestimated, then, the possibility that repeated dosing of BTX-A may induce the formation of antitoxin antibodies, which will void effectiveness [17,18]. Although this event has not yet been described in use on the urinary system, it cannot be excluded that such an event may occur. 

According to our experience, BTX-A in children and adolescents with neurogenic bladder secondary to MMC represents a viable alternative to more invasive procedures, with good objective and subjective results, when anticholinergics are not effective or when their side effects lead the patient to abandon that treatment. The procedure allows also the contemporary treatment of VUR, decreasing the risk of renal damage in children on whom conservative management fails to help. However, the need for additional treatments in the majority of patients is a significant factor in the cost-benefit assessment [19,20,21]. Moreover, another important factor to consider is the compliance of patients to repeated injections that could lead to the interruption of treatment [22]. 

## 5. Conclusions

In conclusion, according to our results, the use of BTX-A appears safe and effective in children and adolescents with neurogenic overactive bladder secondary to MMC with a positive effect on their dryness and quality of life even if its duration and long term reliability needs to be further investigated and standardized.
